# Microbiota in Gut, Oral Cavity, and Mitral Valves Are Associated With Rheumatic Heart Disease

**DOI:** 10.3389/fcimb.2021.643092

**Published:** 2021-03-09

**Authors:** Xue-Rui Shi, Bo-Yan Chen, Wen-Zhen Lin, Yu-Lin Li, Yong-Li Wang, Yan Liu, Jing-Juan Huang, Wei-Wei Zhang, Xiao-Xin Ma, Shuai Shao, Ruo-Gu Li, Sheng-Zhong Duan

**Affiliations:** ^1^ Department of Cardiology, Shanghai Chest Hospital, Shanghai Jiao Tong University, Shanghai, China; ^2^ Laboratory of Oral Microbiota and Systemic Diseases, Shanghai Ninth People’s Hospital, College of Stomatology, Shanghai Jiao Tong University School of Medicine, Shanghai, China; ^3^ National Clinical Research Center for Oral Diseases, Shanghai Key Laboratory of Stomatology & Shanghai Research Institute of Stomatology, Shanghai, China

**Keywords:** rheumatic heart disease, microbiota, gut, subgingival plaque, saliva, mitral valves

## Abstract

Rheumatic heart disease refers to the long-term damage of heart valves and results from an autoimmune response to group A *Streptococcus* infection. This study aimed to analyze the microbiota composition of patients with rheumatic heart disease and explore potential function of microbiota in this disease. First, we revealed significant alterations of microbiota in feces, subgingival plaques, and saliva of the patients compared to control subjects using 16S rRNA gene sequencing. Significantly different microbial diversity was observed in all three types of samples between the patients and control subjects. In the gut, the patients possessed higher levels of genera including *Bifidobacterium* and *Eubacterium*, and lower levels of genera including *Lachnospira*, *Bacteroides*, and *Faecalibacterium*. *Coprococcus* was identified as a super-generalist in fecal samples of the patients. Significant alterations were also observed in microbiota of subgingival plaques and saliva of the patients compared to control subjects. Second, we analyzed microbiota in mitral valves of the patients and identified microbes that could potentially transmit from the gut or oral cavity to heart valves, including *Streptococcus*. Third, we further analyzed the data using random forest model and demonstrated that microbiota in the gut, subgingival plaque or saliva could distinguish the patients from control subjects. Finally, we identified gut/oral microbes that significantly correlated with clinical indices of rheumatic heart disease. In conclusion, patients with rheumatic heart disease manifested important alterations in microbiota that might distinguish the patients from control subjects and correlated with severity of this disease.

## Introduction

Rheumatic heart disease (RHD) is a cardiovascular disease characterized by damages to heart valves, triggered by an autoimmune response to group A *Streptococcus* (GAS) infection (Woldu and Bloomfield, 2016). RHD causes an estimated 300,000 death annually with 30–40 million current cases globally and imposes significant disease burden on low-income countries and some indigenous populations (Watkins et al., 2017). The backbone of RHD therapy is penicillin-based treatment for acute rheumatic fever and replacement of severely damaged heart valves, without an effective vaccine (Tompkins et al., 1972; Nishimura et al., 2014; Woldu and Bloomfield, 2016). There has not been any significant advance in recent history of this field (Watkins et al., 2018). Thus, it is imperative to consider novel strategies for better control of RHD.

Human microbiota has been proven to play essential roles in a wide range of diseases including cardiovascular diseases (Tang et al., 2019). Colon harbors the vast majority of commensal bacteria (Belkaid and Hand, 2014), which can influence immune homeostasis, trigger inflammation, and invade extra-intestinal tissues (Hooper et al., 2012; Anhe et al., 2020). Many studies have focused on revealing the role of gut microbial dysbiosis in the development of cardiovascular diseases such as pulmonary arterial hypertension (Kim et al., 2020), unruptured intracranial aneurysms (Li et al., 2020), chronic heart failure (Kummen et al., 2018), and atherosclerosis (Koren et al., 2010). However, the role of the gut microbiota in RHD has not been studied.

The second most complex population of microbes of human body resides in the oral cavity and influences both oral and systemic health (Zhang et al., 2018; Bui et al., 2019). Periodontal pathogens can alter subgingival microbial composition and host–microorganism interactions, leading to local inflammation and subsequent destruction of periodontal tissues (Hajishengallis et al., 2012). The presence of periodontal bacteria DNA in cardiac tissues and atherosclerotic plaques has suggested connections between oral infections and cardiovascular diseases (Koren et al., 2010; Ziebolz et al., 2018). Periodontitis, a common inflammatory oral disease, increases the risk of adverse pregnancy outcomes, atherosclerosis, stroke, rheumatoid arthritis, diabetes, and other systemic diseases (Pihlstrom et al., 2005). However, there is a paucity of information related the potential role of oral microbiota in RHD.

Here, we aimed to investigate the microbiota composition and structure of RHD patients and explore potential function of microbiota in RHD. We first analyzed microbiota of fecal samples, subgingival plaques and saliva by 16S rRNA gene sequencing to detect alterations between RHD patients and control subjects. We then analyzed microbiota of mitral valves and compared the results with gut and oral microbiota in RHD patients to determine microbial connections between mitral valves and gut/oral cavity. Finally, we explored the possibility using microbiota to discriminate RHD patients from control subjects and analyzed correlations between gut/oral microbiota and severity of RHD.

## Materials and Methods

### Study Cohort

A total of 20 RHD patients and 20 age- and sex-matched control subjects were enrolled in this study. RHD patients with symptomatic severe mitral valvular disease and with a history of acute rheumatic fever were enrolled (Reményi et al., 2012; Nishimura et al., 2017). The exclusion criteria were: inflammatory bowel diseases, irritable bowel syndrome, autoimmune diseases, diarrhea, liver diseases, renal diseases, acute infection, smoking, and use of antibiotics or probiotics 3 months before sample collection.

The study protocol was reviewed and approved by the Human Ethics Committee, Shanghai Chest Hospital, Shanghai Jiaotong University and conducted in accordance with the Principles of Good Clinical Practice and the Declaration of Helsinki. Written informed consent was obtained from all the subjects who participated in the study.

### Sample Collection and DNA Extraction

Fecal samples, subgingival plaques, and saliva were collected from RHD patients and control subjects. Fecal samples were collected in falcon tubes within 24 h of patients’ admission to the hospital and immediately frozen in −80°C. All subjects were instructed to avoid eating, drinking, and use of a toothbrush or mouth rinse 1 hour before sampling of subgingival plaques and saliva. Subgingival plaques were collected using a dental explorer. Saliva was collected and mixed with 2× lysis buffer at a ratio of 1:1. A total of 16 mitral valves were collected during valve replacement surgeries and were immediately frozen in liquid nitrogen under sterile conditions. Samples were kept in sterile containers and stored at −80°C. Bacterial DNA was extracted using Tiangen kits according to the manufacturer’s recommendations and stored at −80°C until further analyses.

### High-Throughput Sequencing and Processing

The V3/V4 regions of 16S rRNA genes were amplified with specific primers of 338F-806R. The PCR products were quantified with PicoGreen dsDNA Assay Kit (Invitrogen, Carlsbad, USA) and sequenced on Illumina Novaseq PE250 platform to generate paired-end reads (2 × 480 bp).

Procession of the sequencing data was performed on QIIME2 platform. Analysis of sequencing data was based on amplicon sequence variants (ASVs) (Bokulich et al., 2018). After chimera detection, high-quality sequences with 97% similarity were clustered into the same ASV. Classification of ASVs was performed based on the Greengenes Database.

### Data Analysis

Richness and *α*-diversity were measured by Chao1 and Shannon index based on the genus profiles. *β*-diversity was visualized using principal coordinate analysis (PCoA) based on the Bray–Curtis distances. ZP-plot was used to identify of key module members (Deng et al., 2012). Within-module connectivity (Zi) and among-module connectivity (Pi) were calculated as previously described (Deng et al., 2012). Microbes were classified into four categories: peripherals (Zi ≤ 2.5, Pi ≤ 0.62), connectors (Zi ≤ 2.5, Pi > 0.62), module hubs (Zi > 2.5, Pi ≤ 0.62), and network hubs (Zi > 2.5, Pi > 0.62) according to the criteria previously set (Deng et al., 2012). Network hubs were considered as super-generalists, which were highly connected within their own modules and among modules (Deng et al., 2012). Raw data of relative abundances of genera were transformed to “log_10_” and Log_10_0 was shown as “−8” in figures.

Linear discriminant analysis effect size (LEfSe) was used to identify features that differed between groups. The threshold of the logarithmic LDA score for discriminative features was set to 3.0. Identified taxa were plotted in cladograms. A random forest classifier was trained to distinguish the two groups based on the genera abundance profile of RHD patients and control subjects. The important genera of the classifier were ranked by Gini index. The performance of the classifier model was evaluated by 10-fold cross-validations and further applied to construct receiver operating characteristic (ROC) curve. The cross-validation accuracy was measured as the area under the ROC curve (AUC). Heatmaps were hierarchically clustered to represent the microbe-clinical indices associations based on the Spearman correlation coefficients.

### Statistics

*α*-diversity was compared using non-parametric Kruskal–Wallis tests and *post hoc* Dunn’s test with FDR correction. *β*-diversity was tested by the ANOSIM method. Raw data of relative abundances of genera were compared using non-parametric Mann–Whitney tests. Correlation analysis was performed using Spearman’s correlation. *Post hoc* power analysis was used to calculate power with significance level set to 0.05 and effect size based on Chao1. The power was 0.88, 0.18 and 0.93 for gut, subgingival plaques and saliva, respectively. Statistical analyses were performed using SPSS 23.0, QIIME2, R package (V3.5.1), or GPower (V3.1.9.4).

## Results

### Alterations of Gut Microbiota in Rheumatic Heart Disease Patients

**Figure 1:**
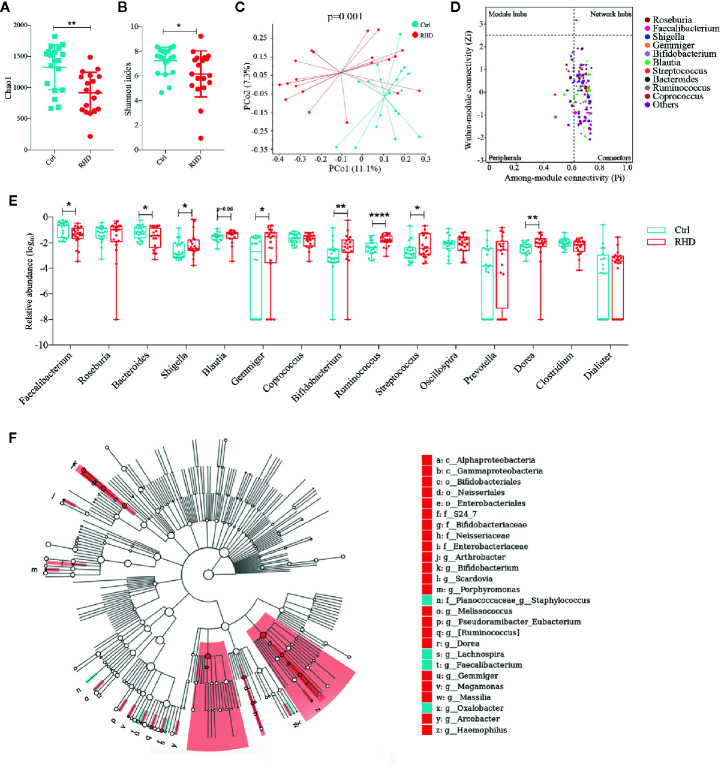
Alterations of gut microbiota in patients with rheumatic heart disease. Microbiota of fecal samples from patients with rheumatic heart disease (RHD) and control subjects (Ctrl) were analyzed using 16S rRNA gene sequencing. **(A)** Richness of fecal microbiota assessed by Chao1. **(B)** *α*-diversity of fecal microbiota assessed by Shannon index. **(C)** *β*-diversity analyzed by principal coordinate analysis (PCoA) based on Bray–Curtis distance of fecal microbiota at genus level. **(D)** Determination of module-based topological roles (peripherals, connectors, module hubs, or network hubs) of fecal microbiota in RHD patients using ZP-plot at genus level. The size of dots represents abundance of each genus. **(E)** Relative abundances of the fifteen most abundant genera in fecal microbiota. **(F)** Taxonomic cladogram of fecal microbiota based on LEfSe. Red color indicates increase and blue indicates decrease of taxa in RHD compared to Ctrl. LDA = 3. Mann–Whitney test was used. n = 20: 20. *P < 0.05; **P < 0.01; ****P < 0.0001.

The demographic data for the enrolled RHD patients and control subjects were summarized in **Supplemental Table 1**. To detect gut microbial alterations, we analyzed the microbiota in fecal samples of control subjects and RHD patients using 16S rRNA gene sequencing. Gut microbiota of RHD patients showed significantly lower richness illustrated by Chao1 and *α*-diversity (within-sample diversity) illustrated by Shannon index compared to those of control subjects (**Figures 1A, B**). Principal coordinate analysis (PCoA) based on Bray–Curtis distance was performed to determine *β*-diversity (between-sample diversity) of gut microbiota, which demonstrated significant difference between RHD patients and control subjects (**Figure 1C**). To understand interactions among different microbes in gut microbiota of RHD patients, ZP-plot was used to analyze topological roles, and *Coprococcus* was identified as a super-generalist (shown as network hubs) (**Figure 1D**). Among the 15 most abundant genera, relative abundance of *Faecalibacterium* and *Bacteroides* significantly decreased, while that of *Shigella*, *Gemmiger*, *Bifidobacterium*, *Ruminococcus*, *Streptococcus* and *Dorea* significantly increased in gut microbiota of RHD patients compared to control subjects (**Figure 1E**). In addition, relative abundance of *Blauita* showed a trend of increase without statistical significance in gut microbiota of RHD patients (**Figure 1E**). We next employed linear discriminant analysis effect size (LEfSe) to identify taxa that discriminate microbial composition between disease states. LEfSe identified higher levels of taxa including Arthrobacter, *Bifidobacterium*, *Porphyromonas*, *Melissococccus*, *Eubacterium*, *Ruminococcus*, *Dorea*, *Gemmiger*, *etc*., as well as lower levels of taxa such as *Staphylococcus*, *Lachnospira*, *Faecalibacterium*, and *Oxalobacter* in fecal samples of RHD patients (**Figure 1F**). These results demonstrated considerable changes of the gut microbiota in RHD patients compared to control subjects.

### Alterations of Subgingival Plaque Microbiota in Rheumatic Heart Disease Patients

**Figure 2:**
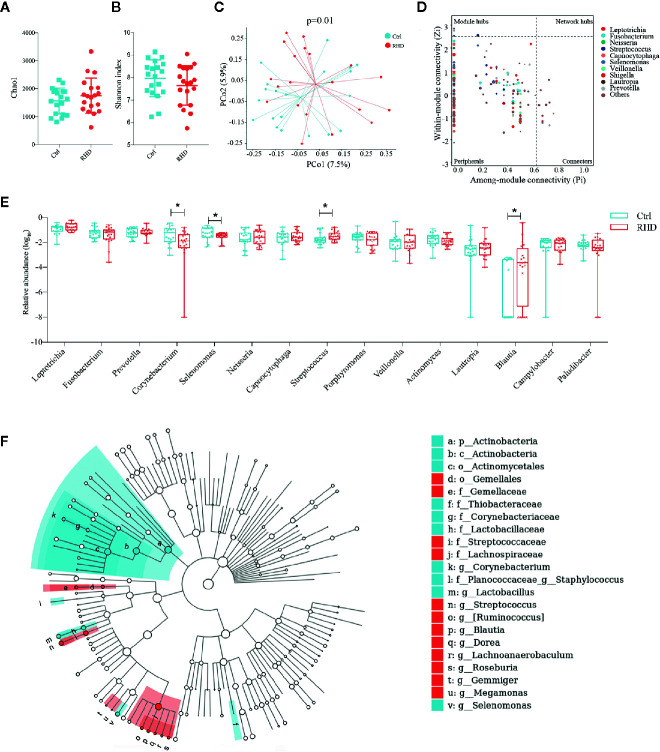
Alterations of subgingival plaque microbiota in patients with rheumatic heart disease. Microbiota of subgingival plaques (SP) from patients with rheumatic heart disease (RHD) and control subjects (Ctrl) were analyzed using 16S rRNA gene sequencing. **(A)** Richness of SP microbiota assessed by Chao1. **(B)** *α*-diversity of SP microbiota assessed by Shannon index. **(C)** *β*-diversity analyzed by principal coordinate analysis (PCoA) based on Bray–Curtis distance of SP microbiota at genus level. **(D)** Determination of module-based topological roles (peripherals, connectors, module hubs, or network hubs) of SP microbiota in RHD patients using ZP-plot at genus level. The size of dots represents abundance of each genus. **(E)** Relative abundances of the fifteen most abundant genera in SP microbiota. **(F)** Taxonomic cladogram of SP microbiota based on LEfSe. Red color indicates increase and blue indicates decrease of taxa in RHD compared to Ctrl. LDA = 3. Mann–Whitney test was used. n = 20: 20. *P < 0.05.

Next we analyzed the microbiota in subgingival plaque samples of control subjects and RHD patients using 16S rRNA gene sequencing. Subgingival plaque microbiota showed no differences of richssness and *α*-diversity between the two groups illustrated by Chao1 and Shannon index (**Figures 2A, B**). Results of PCoA based on Bray–Curtis distance demonstrated moderate difference in *β*-diversity between RHD patients and control subjects (**Figure 2C**). There was no super-generalist based on modular topological roles in subgingival plaque microbiota of RHD patients (**Figure 2D**). Among the 15 most abundant genera, relative abundance of *Corynebacterium* and *Selenomonas* significantly decreased, while that of *Streptococcus* and *Blautia* significantly increased in subgingival plaque microbiota of RHD patients compared to control subjects (**Figure 2E**). LEfSe identified higher levels of taxa including *Streptococcus*, *Ruminococcus*, *Blautia*, *Dorea*, *Lachnoanaerobaculum*, *Roseburia*, *Gemmiger*, as well as lower levels of taxa such as *Corynebacterium*, *Staphylococcus*, *Lactobacillus* and *Selenomonas* in subgingival plaque samples of RHD patients (**Figure 2F**).

### Alterations of Salivary Microbiota in Rheumatic Heart Disease Patients

**Figure 3:**
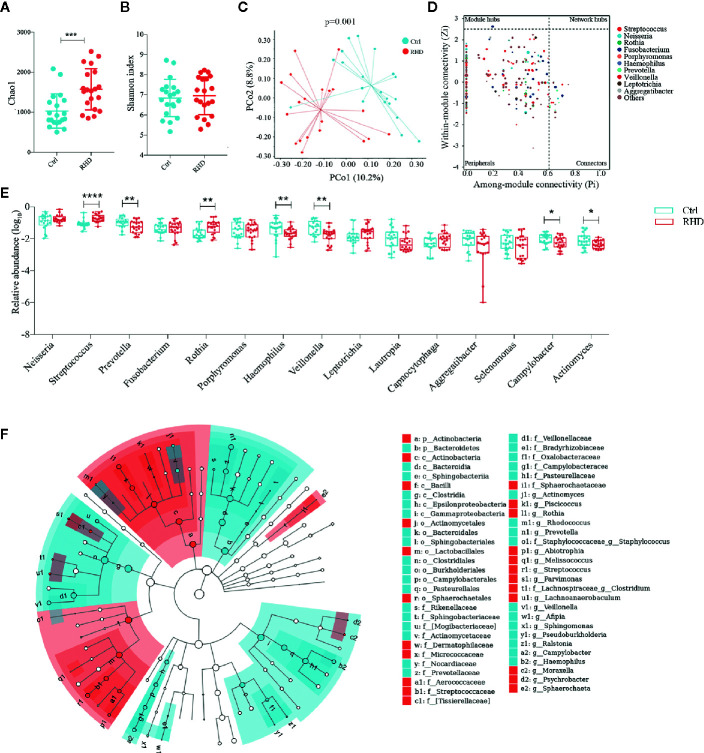
Alterations of salivary microbiota in patients with rheumatic heart disease. Microbiota of saliva from patients with rheumatic heart disease (RHD) and control subjects (Ctrl) were analyzed using 16S rRNA gene sequencing. **(A)** Richness of salivary microbiota assessed by Chao1. **(B)** *α*-diversity of salivary microbiota assessed by Shannon index. **(C)** *β*-diversity analyzed by principal coordinate analysis (PCoA) based on Bray–Curtis distance of salivary microbiota at genus level. **(D)** Determination of module-based topological roles (peripherals, connectors, module hubs, or network hubs) of salivary microbiota in RHD patients using ZP-plot at genus level. The size of dots represents abundance of each genus. **(E)** Relative abundances of the fifteen most abundant genera in salivary microbiota. **(F)** Taxonomic cladogram of salivary microbiota based on LEfSe. Red color indicates increase and blue indicates decrease of taxa in RHD compared to Ctrl. LDA = 3. Mann–Whitney test was used. n = 20: 20. *P < 0.05; **P < 0.01; ***P < 0.001; ****P < 0.0001.

We further analyzed microbiota in saliva of control subjects and RHD patients using 16S rRNA gene sequencing. Salivary microbiota of RHD patients showed significantly higher richness illustrated by Chao1 and similar *α*-diversity illustrated by Shannon index compared to those of control subjects (**Figures 3A, B**). Results of PCoA based on Bray–Curtis distance demonstrated significantly different *β*-diversity between RHD patients and control subjects (**Figure 3C**). There was no super-generalist based on modular topological roles in salivary microbiota of RHD patients (**Figure 3D**). Among the 15 most abundant genera, relative abundance of *Prevotella*, *Haemophilus*, *Veillonella*, *Campylobacter*, and *Actinomyces* significantly decreased, while that of *Streptococcus* and *Rothia* significantly increased in salivary microbiota of RHD patients compared to control subjects (**Figure 3E**). LEfSe identified higher levels of taxa including *Piscicoccus*, *Rothia*, *Abiotrophia*, *Melissococcus*, *Streptococcus*, *Parvimonas*, *Clostridium*, *Lachnoanaerobaculum*, *etc*., as well as lower levels of taxa such as *Actinomyces*, *Rhodococcus*, *Prevotella*, *Staphylococcus*, *Veillonella*, *Afipia*, *Sphingomonas*, *etc*. (**Figure 3F**).

### Comparisons of Microbiota Composition Between Mitral Valves and Other Body Sites in Rheumatic Heart Disease Patients

**Figure 4:**
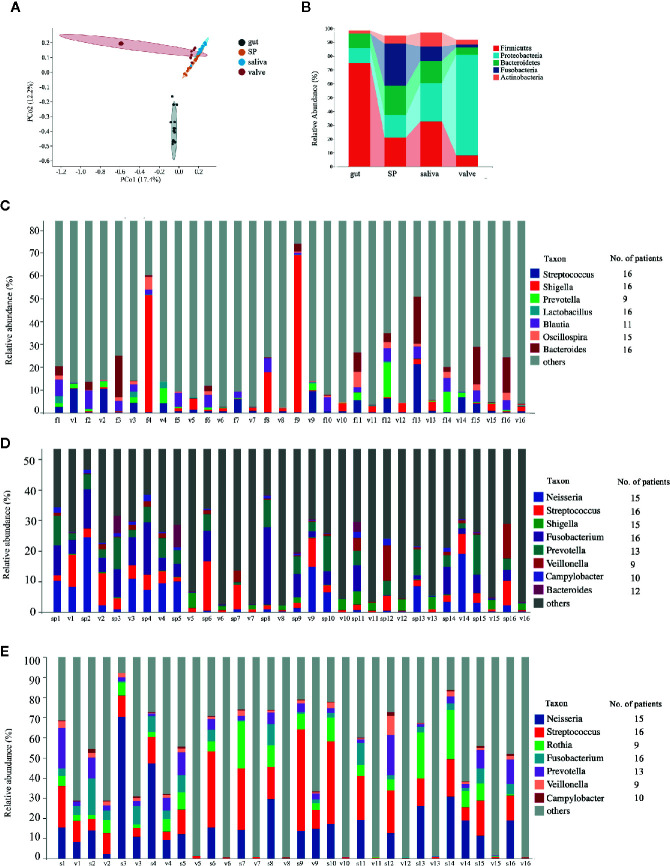
Comparisons of microbiota composition between mitral valves and other body sites of patients with rheumatic heart disease. Microbiota of mitral valves from patients with rheumatic heart disease (RHD) were analyzed using 16S rRNA gene sequencing. **(A)** Comparison of microbial *β*-diversity (PCoA) among gut, subgingival plaques (SP), saliva, and mitral valves (valve) of RHD patients. **(B)**Taxonomic composition of microbiota at different body sites of RHD patients at phylum level. **(C)** Illustration of genera detected in both fecal sample (f) and mitral valve (v) of each RHD patient. The numbers (1–16) denote RHD patients. The column on right indicates the number of patients who possess the corresponding genus in both sites. **(D)** Illustration of genera detected in both subgingival plaque (sp) and mitral valve (v) of each RHD patient. **(E)** Illustration of genera detected in both saliva (s) and mitral valve (v) of each RHD patient. n = 16.

Subsequently, we analyzed the microbiota in mitral valves of RHD patients (n = 16) using 16S rRNA gene sequencing. The 15 most abundant genera in mitral valves of RHD patients were *Ralstonia*, *Pelomonas*, *Acinetobacter*, *Neisseria*, *Sphingomonas*, *Streptococcus*, *Agrobacterium*, *Thermus*, *Shigella*, *Rothia*, *Fusobacterium*, *Prevotella*, *Afipia*, *Caulobacter*, and *Burkholderia* (**Supplemental Figure 1A**). There was no super-generalist based on modular topological roles in microbiota of mitral valves of RHD patients (**Supplemental Figure 2**). We next compared microbiota in mitral valves with gut and oral microbiota in these 16 RHD patients. PCoA based on Bray–Curtis distance demonstrated distinct *β*-diversity among microbiota of mitral valves, gut, and oral cavity, although microbiota of mitral valves in some RHD patients had similar *β*-diversity with oral microbiota (**Figure 4A**). As expected, *β*-diversity of subgingival plaque microbiota and salivary microbiota are similar (**Figure 4A**). Taxonomic composition plots at phylum level showed that mitral valves contained significantly more *Proteobacteria* and less *Firmicutes* compared to gut, subgingival plaques, and saliva (**Figure 4B**), similar to the results of a previous study in atherosclerotic plaques (Koren et al., 2010). We further searched for microbes shared by mitral valves and other body sites using three criteria: (1) detected in mitral valve and at least another body site of an individual RHD patient; (2) detected in mitral valves and another body site of more than eight RHD patients (50%) simultaneously; (3) relative abundance ranked among top 35 (**Supplemental Figure 1**). Mitral valves and fecal samples shared *Streptococcus*, *Shigella*, *Lactobacillus*, and *Bacteroides* in all 16 patients, *Oscillospira* in 15 patients, *Blautia* in 11 patients, and *Prevotella* in nine patients (**Figure 4C**). Mitral valves and subgingival plaques shared *Streptococcus* and *Fusobacterium* in all 16 patients, *Neisseria* and *Shigella* in 15 patients, Prevotella in 13 patients, *Bacteroides* in 12 patients, *Campylobacter* in 10 patients, and *Veillonella* in nine patients (**Figure 4D**). Mitral valves and saliva shared *Streptococcus* and *Fusobacterium* in all 16 patients, *Neisseria* in 15 patients, *Prevotella* in 13 patients, *Campylobacter* in 10 patients, as well as *Rothia* and *Veillonella* in nine patients (**Figure 4E**).

### Microbiota Discriminates Rheumatic Heart Disease Patients From Control Subjects

**Figure 5:**
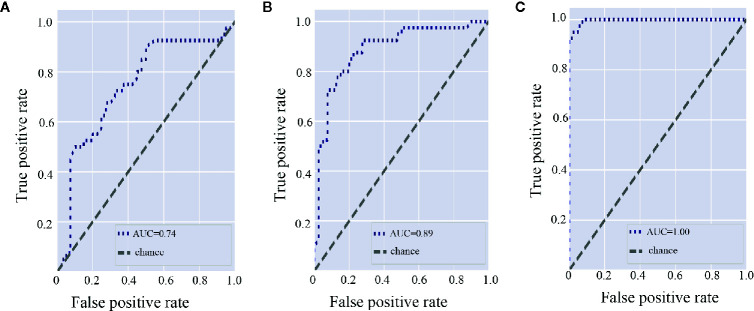
Gut and oral microbiota differentiate rheumatic heart disease patients from control subjects. **(A)** Receiver operating characteristic (ROC) curve according to random forest model for fecal microbiota. A greater area under the ROC curve (AUC) indicates better performance. **(B)** ROC curve according to random forest model for subgingival plaque microbiota. **(C)** ROC curve according to random forest model for salivary microbiota.

To explore the diagnostic value of microbiota in discriminating RHD patients from healthy controls, we constructed random forest classifiers. According to random forest classifier based on gut microbiota, the top 15 important gut genera were *Ruminococcus*, *Dorea*, *Arthrobacter*, *Lachnospira*, *Shigella*, *Gemmiger*, *Bifidobacterium*, *Ralstonia*, *Faecalibacterium*, *Subdoligranulum*, *Burkholderia*, *Blautia*, *Turicibacter*, *Streptococcus*, and *Selenomonas* (**Supplemental Figure 3A**). Area under the curve (AUC) of the gut microbiota was 0.74 (**Figure 5A**). As for subgingival plaque microbiota, the top 15 important subgingival plaque genera were *Roseburia*, *Acinetobacter*, *Turicibacter*, *Bifidobacterium*, *Lactobacillus*, *Leptotrichia*, *Faecalibacterium*, *Lachnoanaerobaculum*, *Clostridium*, *Streptococcus*, *Atopobium*, *Blautia*, *Gemmiger*, *Filifactor*, and *Granulicatella* (**Supplemental Figure 3B**). AUC of subgingival plaque microbiota was 0.89 (**Figure 5B**). In saliva, the top 15 important salivary genera were *Streptococcus*, *Shigella*, *Rothia*, *Pelomonas*, *Lachnoanaerobaculum*, *Parvimonas*, *Arthrobacter*, *Clostridium*, *Abiotrophia*, *Afipia*, *Streptobacillus*, *Ralstonia*, *Turicibacter*, *Cardiobacterium*, and *Haemophilus* (**Supplemental Figure 3C**). AUC of salivary microbiota was 1.00 (**Figure 5C**).

### Microbial Taxa Correlate With Clinical Indices in Rheumatic Heart Disease Patients

**Figure 6:**
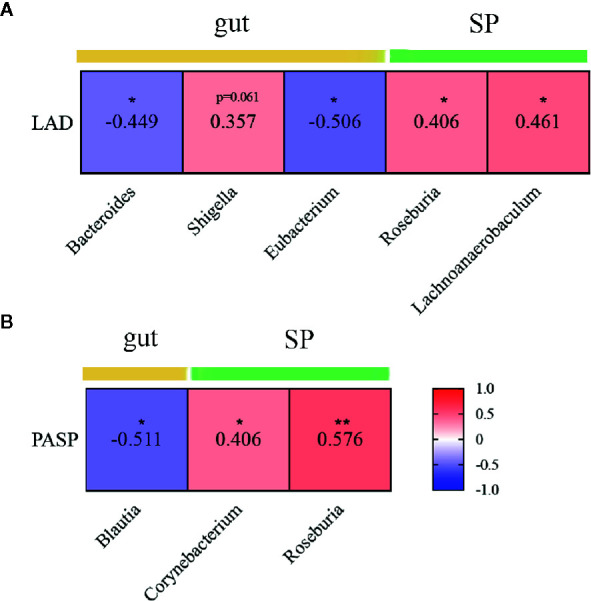
Correlations between microbial genera and clinical indices in patients with rheumatic heart disease. **(A)** Heatmap showing correlations between different genera and LAD. **(B)** Heatmap showing correlations between different genera and PASP. LAD indicates left atrial diameter; and PASP, pulmonary artery systolic pressure. SP indicates subgingival plaque. Spearman’s rank correlation coefficients are illustrated in squares. *P < 0.05; **P < 0.01.

Mitral valves are the most common heart valves affected by RHD (Russell et al., 2017). Mitral stenosis and regurgitation cause depressed left atrial compliance and elevated left atrial pressure, leading to atrial remodelling and increased left atrial diameter (LAD). Furthermore, pulmonary hypertension is one of the most frequent medical complications in RHD (Watkins et al., 2018). We explored the relationship between these clinical indices and microbial taxa differentially enriched or important to discriminate RHD patients from healthy controls. The results showed that *Bacteroides* and *Eubacterium* in the gut negatively correlated with LAD; *Roseburia* and *Lachnoanaerobaculum* in subgingival plaques, on the other hand, positively correlated with LAD (**Figure 6A**). *Blautia* in the gut negatively correlated with pulmonary artery systolic pressure (PASP); *Corynebacterium* and *Roseburia* in subgingival plaques, on the other hand, positively correlated with PASP (**Figure 6B**).

## Discussion

To our knowledge, this work is the first one to present comprehensive characterization of the microbiota in RHD patients. Our study demonstrated that RHD patients had altered gut and oral microbiota, which was likely to translocate to mitral valves and correlate with severity of the disease. These results provided new insights on etiology, diagnosis, prevention, and treatment of RHD.

We identified significant alterations in the microbial profile of gut in RHD patients. We observed that relative abundances of *Bifidobacterium* and *Eubacterium* increased and those of *Faecalibacterum* and *Bacteroides* decreased in RHD patients. Moreover, *Coprococcus* served as a super-generalist in gut microbiota of RHD patients. *Bifidobacterium* has been considered a probiotic that can alter host microbiota in favor of a “healthier” composition (Toscano et al., 2017). *Eubacterium*, *Faecalibacterum*, *Bacteroides*, *Coprococcus*, and *Blautia* are important propionate- or butyrate-producing bacteria (Hoyles and Swann, 2019; Blaak et al., 2020). Propionate can enhance generation of macrophage and dendritic cell precursors in the bone marrow (Trompette et al., 2014) and butyrate can facilitate extrathymic generation of T-reg cells (Arpaia et al., 2013). Butyrate also helps to maintain the integrity of the intestinal epithelium and reduce injury in distant organs such as lungs (Haak et al., 2018; Hoyles and Swann, 2019). Therefore, it seemed counterintuitive that *Bifidobacterium* and *Eubacterium* increased in the gut microbiota of RHD patients. We postulated that during the progression of RHD the decrease of some beneficial genera such as *Faecalibacterum* and *Bacteroides* contributed to disruption of immune homeostasis, resulting in passive increase of some other beneficial genera such as *Bifidobacterium* and *Eubacterium* in response to lasting immune dysbiosis. Similarly, previous studies have demonstrated an increase of *Bifidobacterium* or *Eubacterium* in patients with inflammatory bowel disease (Wang et al., 2013) or systemic lupus erythematosus (He et al., 2016). We also observed that *Bacteroides*, *Eubacterium*, and *Blautia* in the gut negatively correlated with severity of RHD, suggesting beneficial roles of these three genera in RHD. However, neither *Bifidobacterium* nor *Faecalibacterum* significantly correlated with any clinical indices of RHD (Data not shown). The detailed functions and mechanisms of these individual microbes in RHD remain to be further delineated.

The gut microbiota of RHD patients manifested some similarities but more differences comparing to that of patients with other diseases. We observed increases in most of the abundant genera in the gut microbiota of RHD patients. The reason may be that RHD patients were subjected to high levels of stress and gut inflammation that reduced gastrointestinal motility and clearance capacity, leading to bacterial overgrowth in turn. This phenomenon has been previously observed in patients with type 1 diabetes, chronic heart failure, or other critical illness (Btaiche et al., 2010; Pasini et al., 2016; Malik et al., 2018). The gut microbial profile of RHD patients differed significantly from other cardiovascular diseases such as chronic heart failure and pulmonary arterial hypertension (Kummen et al., 2018; Kim et al., 2020). For example, gut microbiota of patients with chronic heart failure had relatively low abundance in *Bifidobacterium* and high in *Prevotella*  (Kummen et al., 2018), whereas our results demonstrated that the gut microbiota of RHD patients had relatively high abundance in *Bifidobacterium* and no difference in *Prevotella*. Some genera in RHD patients had similar alterations as in patients with pulmonary arterial hypertension, including decreased *Bacteroides* and increased *Bifidobacterium* (Kim et al., 2020). However, alterations of some other genera were completely different between RHD patients and patients with pulmonary arterial hypertension. For example, the abundance of *Eubacterium* increased in RHD patients, while it decreased in patients with pulmonary arterial hypertension (Kim et al., 2020). Thus, we propose that the same bacteria may respond to different stimuli specifically and play different roles in various disease conditions. Further animal experiments should be implemented to confirm their specific functions. It is the unique microbial profile that makes it possible to discriminate RHD patients from healthy controls or even other diseases.

We also identified significant microbial alterations in oral cavity of RHD patients. A large variety of microbiota resides in different locations of the oral cavity (Avila et al., 2009). We analyzed the microbiota of saliva and subgingival plaques in this study. Salivary microbiota of RHD patients showed higher richness than that of control subjects, likely because of poor oral hygiene (Maharaj and Vayej, 2012; Belstrom et al., 2018). The difference in *β*-diversity of salivary microbiota between RHD and control subjects was more dramatic in comparison with that of gut and subgingival plaque microbiota. In line with this, the diagnostic value of salivary microbiota was the greatest according to random forest analysis. The results underpinned the theory that saliva represented a significant source of discriminatory biomarkers for oral and systemic diseases (Yoshizawa et al., 2013). For example, salivary microbes were able to correlate with clinical indices and stratify active and moderately active patients of rheumatoid arthritis (Zhang et al., 2015). Similar results were obtained in Crohn’s disease, another autoimmune disease (Zhang et al., 2020). Although microbes in saliva had large potential to differentiate RHD from control subjects, there was no correlation between any single salivary microbe and RHD severity. The reason may be that salivary microbes closely interacted with each other and functioned as a whole (Jenkinson and Lamont, 2005). Our data showed that genus such as *Roseburia*, *Lachnoanaerobaculum*, and *Corynebacterium* in subgingival plaques correlated with RHD severity, although the functions of these genera in periodontal disease or RHD remain to be further explored.

Our results also showed that *Streptococcus* significantly increased in saliva of RHD patients. There are 74 species under the genus of *Streptococcus* (Wong and Yuen, 2012). GAS is responsible for pharyngitis and the post-infection sequela, including RHD (Soderholm et al., 2018). GAS can survive the oral immune defense system and remain viable for long periods (Walker et al., 2014). Not all *Streptococcus* spp. are harmful. For instance, *Streptococcus salivarius* K12 is a probiotic intended for use in the oral cavity and can antagonize the growth of GAS (Di Pierro et al., 2016). Therefore, it needs to be further determined which species of *Streptococcus* contributed to the changes we observed in the oral microbiota of RHD patients. *Streptococcus* also increased in subgingival plaques of RHD patients, likely a consequence of the increase of this genus in saliva (Avila et al., 2009).

Our data suggested potential transmission of microbes from the gut or oral cavity to heart valves of RHD patients. Some genera were shared by mitral valves and gut or oral cavity in RHD patients, indicating that microbes in the gut and/or oral cavity might translocate to the mitral valves. In the context of immunological cascade caused by GAS infection, gut microbial dysbiosis may increase endotoxin production and weaken gut barrier function, leading to increase of intestinal permeability and subsequent bacterial translocation (Camilleri, 2019). Oral cavity is another potential origin of the microbiota in mitral valves. Transmission of oral microbes to other body sites can be caused by invasive dental procedure or even normal daily activities such as tooth brushing and food intake (Smith and Nehring, 2020). Our results demonstrated that the microbiota in mitral valves partially overlapped with that in oral cavity. Intriguingly, *Campylobacter* was distributed in most mitral valves and oral cavity but only presented in one fecal sample of RHD patients, pointing to the possibility of oral-to-valve but not gut-to-valve translocation of this microbe. *Streptococcus* was abundantly distributed in mitral valves of every RHD patient. This is consistent with the theory that GAS or its components can enter the circulation and gain access to the subendothelial collagen matrix (Tandon et al., 2013). *Neisseria, Lactobacillus, Campylobacter and Prevotella* were detected in the mitral valves of most RHD patients. It would be reasonable to speculate that these microbes, together with *Streptococcus*, could translocate from the gut and/or oral cavity to the mitral valves, creating antigens that provoke autoimmune response against host cardiac tissues over the progression of RHD.

Our data provided new insights on the etiology, diagnosis, prevention, and treatment of RHD. First, unique microbial profiles of RHD broadened the concept of genetic susceptibility to RHD. Only a minority (1–2%) of populations living in GAS-endemic areas develop RHD (Carapetis et al., 2000) and specific genetic markers have been linked to RHD (Gray et al., 2017). Microbiota, considered as the second genome of the human body, may play a role in genetic predisposition to RHD (Grice and Segre, 2012). Second, unique microbial profiles may serve as a supplemental diagnostic tool for RHD patients. Our results illustrated that it was feasible to differentiate RHD patients from control subjects using microbiota. Third, our data suggested that the microbiota played important roles in RHD treatment. Broad-spectrum antibiotics may eliminate beneficial microbes and sometimes cause secondary infections (Kelly and LaMont, 2008). Antibiotics designed with the narrowest spectra targeting GAS or regular use of probiotics may reduce the ecologically undesirable side effects of non-discriminative antibiotic chemoprophylaxis (Lemon et al., 2012). At last, the microbiota may serve as a viable therapeutic target for halting progression to severe mitral stenosis and for post-surgery management. The microbiota may influence immune homeostasis, trigger inflammation, invade tissues, and create antigens (Hooper et al., 2012; Tandon et al., 2013; Anhe et al., 2020). It is feasible to treat diseases through manipulation of the microbiota. For example, fecal microbiota transplantation has been safely performed in the treatment of diarrhea and sepsis in critical care (Li et al., 2015). Moreover, microbiota affects drug pharmacokinetics. The rate of absorption and bioavailability of many oral drugs depends on their exposure to bacterial enzymes before entering the circulation (Li and Jia, 2013). For RHD patients with prosthetic valves, the gut microbiota has been implicated to affect anticoagulant therapy that is mandatory after surgery (Wang et al., 2020). Our study is a start point for potential microbiota-targeting therapies, although more studies are required to establish causative links between the microbiota and RHD.

In conclusion, we described important alterations in the microbiota of RHD patients, provided evidence that microbiota in gut and oral cavity might translocate to mitral valves, demonstrated the possibility of distinguishing patients from control subjects using microbiota, and identified gut/oral microbes that correlated with severity of RHD. Our study paved a promising path for using microbiota as a potential diagnostic, prophylactic, and therapeutic tool for RHD.

## Data Availability Statement

The datasets presented in this study can be found in online repositories. The names of the repository/repositories and accession number(s) can be found below: https://www.ncbi.nlm.nih.gov; PRJNA682705.

## Ethics Statement

The studies involving human participants were reviewed and approved by the Human Ethics Committee, Shanghai Chest Hospital, Shanghai Jiaotong University, and conducted in accordance with the Principles of Good Clinical Practice and the Declaration of Helsinki. The patients/participants provided their written informed consent to participate in this study.

## Author Contributions

R-GL and S-ZD designed and supervised the project. X-RS and B-YC performed the statistical analyses. W-ZL, Y-LL, Y-LW, J-JH, and W-WZ collected the clinical samples and extracted the DNA. YL, X-XM, and SS interpreted the data. X-RS wrote the manuscript. R-GL and S-ZD read and revised the manuscript. All authors contributed to the article and approved the submitted version.

## Funding

This work was supported by grants from the National Natural Science Foundation of China (81991500, 81991503, 81921002, 82070262), Shanghai’s Top Priority Clinical Medicine Center (2017ZZ01011), Science and Technology Commission of Shanghai Municipality (19140905302), Shanghai Shen Kang Hospital Development Center Clinical Research Plan of SHDC (2020CR3026B), and the Innovative Research Team of High-Level Local Universities in Shanghai (SSMU-ZDCX20180900).

## Conflict of Interest

The authors declare that the research was conducted in the absence of any commercial or financial relationships that could be construed as a potential conflict of interest.

## References

[B1] Anhe F. F. Jensen B. A. H. Varin T. V. Servant F. Van Blerk S. Richard D.. (2020). Type 2 diabetes influences bacterial tissue compartmentalisation in human obesity. Nat. Metab. 2 (3), 233–242. 10.1038/s42255-020-0178-9 32694777

[B2] Arpaia N. Campbell C. Fan X. Y. Dikiy S. van der Veeken J. deRoos P.. (2013). Metabolites produced by commensal bacteria promote peripheral regulatory T-cell generation. Nature 504 (7480), 451–45+. 10.1038/nature12726 24226773 PMC3869884

[B3] Avila M. Ojcius D. M. Yilmaz O. (2009). The Oral Microbiota: Living with a Permanent Guest. DNA Cell Biol. 28 (8), 405–411. 10.1089/dna.2009.0874 19485767 PMC2768665

[B4] Belkaid Y. Hand T. W. (2014). Role of the Microbiota in Immunity and Inflammation. Cell 157 (1), 121–141. 10.1016/j.cell.2014.03.011 24679531 PMC4056765

[B5] Belstrom D. Sembler-Moller M. L. Grande M. A. Kirkby N. Cotton S. L. Paster B. J.. (2018). Impact of Oral Hygiene Discontinuation on Supragingival and Salivary Microbiomes. JDR Clin. Trans. Res. 3 (1), 57–64. 10.1177/2380084417723625 29662960 PMC5896869

[B6] Blaak E. E. Canfora E. E. Theis S. Frost G. Groen A. K. Mithieux G.. (2020). Short chain fatty acids in human gut and metabolic health. Benef. Microbes 11 (5), 411–455. 10.3920/BM2020.0057 32865024

[B7] Bokulich N. A. Kaehler B. D. Rideout J. R. Dillon M. Bolyen E. Knight R.. (2018). Optimizing taxonomic classification of marker-gene amplicon sequences with QIIME 2 ‘ s q2-feature-classifier plugin. Microbiome 6 (1), 90. 10.1186/s40168-018-0470-z 29773078 PMC5956843

[B8] Btaiche I. F. Chan L. N. Pleva M. Kraft M. D. (2010). Critical Illness, Gastrointestinal Complications, and Medication Therapy during Enteral Feeding in Critically Ill Adult Patients. Nutr. Clin. Pract. 25 (1), 32–49. 10.1177/0884533609357565 20130156

[B9] Bui F. Q. Coutinho Almeida-da-Silva C. L. Huynh B. Trinh A. Liu J. Woodward J.. (2019). Association between periodontal pathogens and systemic disease. Biomed. J. 42 (1), 27–35. 10.1016/j.bj.2018.12.001 30987702 PMC6468093

[B10] Camilleri M. (2019). Leaky gut: mechanisms, measurement and clinical implications in humans. Gut 68 (8), 1516–1526. 10.1136/gutjnl-2019-318427 31076401 PMC6790068

[B11] Carapetis J. R. Currie B. J. Mathews J. D. (2000). Cumulative incidence of rheumatic fever in an endemic region: a guide to the susceptibility of the population? Epidemiol. Infect. 124 (2), 239–244. 10.1017/s0950268800003514 10813149 PMC2810907

[B12] Deng Y. Jiang Y. H. Yang Y. He Z. Luo F. Zhou J. (2012). Molecular ecological network analyses. BMC Bioinf. 13, 113. 10.1186/1471-2105-13-113 PMC3428680 22646978

[B13] Di Pierro F. Colombo M. Zanvit A. Rottoli A. S. (2016). Positive clinical outcomes derived from using Streptococcus salivarius K12 to prevent streptococcal pharyngotonsillitis in children: a pilot investigation. Drug Healthcare Patient Saf. 8, 77–81. 10.2147/dhps.S117214 PMC5123729 27920580

[B14] Gray L.-A. D’Antoine H. A. Tong S. Y. C. McKinnon M. Bessarab D. Brown N.. (2017). Genome-Wide Analysis of Genetic Risk Factors for Rheumatic Heart Disease in Aboriginal Australians Provides Support for Pathogenic Molecular Mimicry. J. Infect. Dis. 216 (11), 1460–1470. 10.1093/infdis/jix497 29029143

[B15] Grice E. A. Segre J. A. (2012). The human microbiome: our second genome. Annu Rev Genomics Hum Genet (2012) 13, 151–170. 10.1146/annurev-genom-090711-163814 22703178 PMC3518434

[B16] Haak B. W. Littmann E. R. Chaubard J.-L. Pickard A. J. Fontana E. Adhi F.. (2018). Impact of gut colonization with butyrate-producing microbiota on respiratory viral infection following allo-HCT. Blood 131 (26), 2978–2986. 10.1182/blood-2018-01-828996 29674425 PMC6024637

[B17] Hajishengallis G. Darveau R. P. Curtis M. A. (2012). The keystone-pathogen hypothesis. Nat. Rev. Microbiol. 10 (10), 717–725. 10.1038/nrmicro2873 22941505 PMC3498498

[B18] He Z. Shao T. Li H. Xie Z. Wen C. (2016). Alterations of the gut microbiome in Chinese patients with systemic lupus erythematosus. Gut Pathog. 8 (1), 64. 10.1186/s13099-016-0146-9 27980687 PMC5146896

[B19] Hooper L. V. Littman D. R. Macpherson A. J. (2012). Interactions Between the Microbiota and the Immune System. Science 336 (6086), 1268–1273. 10.1126/science.1223490 22674334 PMC4420145

[B20] Hoyles L. Swann J. (2019). “Influence of the Human Gut Microbiome on the Metabolic Phenotype,” in The Handbook of Metabolic Phenotyping (Elsevier Press), 535–560.

[B21] Jenkinson H. F. Lamont R. J. (2005). Oral microbial communities in sickness and in health. Trends Microbiol. 13 (12), 589–595. 10.1016/j.tim.2005.09.006 16214341

[B22] Kelly C. P. LaMont J. T. (2008). Clostridium difficile - More difficult than ever. N. Engl. J. Med. 359 (18), 1932–1940. 10.1056/NEJMra0707500 18971494

[B23] Kim S. Rigatto K. Gazzana M. B. Knorst M. M. Richards E. M. Pepine C. J.. (2020). Altered Gut Microbiome Profile in Patients With Pulmonary Arterial Hypertension. Hypertension 75 (4), 1063–1071. 10.1161/HYPERTENSIONAHA.119.14294 32088998 PMC7067661

[B24] Koren O. Spor A. Felin J. Fak F. Stombaugh J. Tremaroli V.. (2010). Human oral, gut, and plaque microbiota in patients with atherosclerosis. Proc. Natl. Acad. Sci. 108 (Supplement_1), 4592–4598. 10.1073/pnas.1011383107 20937873 PMC3063583

[B25] Kummen M. Mayerhofer C. C. K. Vestad B. Broch K. Awoyemi A. Storm-Larsen C.. (2018). Gut Microbiota Signature in Heart Failure Defined From Profiling of 2 Independent Cohorts. J. Am. Coll. Cardiol. 71 (10), 1184–1186. 10.1016/j.jacc.2017.12.057 29519360

[B26] Lemon K. P. Armitage G. C. Relman D. A. Fischbach M. A. (2012). Microbiota-Targeted Therapies: An Ecological Perspective. Sci. Trans. Med. 4 (137), 137rv135. 10.1126/scitranslmed.3004183 PMC5725196 22674555

[B27] Li H. Jia W. (2013). Cometabolism of Microbes and Host: Implications for Drug Metabolism and Drug-Induced Toxicity. Clin. Pharmacol. Ther. 94 (5), 574–581. 10.1038/clpt.2013.157 23933971

[B28] Li Q. Wang C. Tang C. He Q. Zhao X. Li N.. (2015). Successful treatment of severe sepsis and diarrhea after vagotomy utilizing fecal microbiota transplantation: a case report. Crit. Care 19 (1), 37 10.1186/s13054-015-0738-7 25881250 PMC4346118

[B29] Li H. Xu H. Li Y. Jiang Y. Hu Y. Liu T.. (2020). Alterations of gut microbiota contribute to the progression of unruptured intracranial aneurysms. Nat. Commun. 11 (1), 328. 10.1038/s41467-020-16990-3 32587239 PMC7316982

[B30] Maharaj B. Vayej A. C. (2012). Oral health of patients with severe rheumatic heart disease. Cardiovasc. J. Afr. 23 (6), 336–339. 10.5830/cvja-2012-009 22836156 PMC3734880

[B31] Malik A. Morya R. K. Bhadada S. K. Rana S. (2018). Type 1 diabetes mellitus: Complex interplay of oxidative stress, cytokines, gastrointestinal motility and small intestinal bacterial overgrowth. Eur. J. Clin. Invest. 48 (11), e13021. 10.1111/eci.13021 30155878

[B32] Nishimura R. A. Otto C. M. Bonow R. O. Carabello B. A. Erwin J. P. Guyton R. A.. (2014). 2014 AHA/ACC Guideline for the Management of Patients With Valvular Heart Disease: Executive Summary. J. Am. Coll. Cardiol. 63 (22), 2438–2488. 10.1016/j.jacc.2014.02.537 24603192

[B33] Nishimura R. A. Otto C. M. Bonow R. O. Carabello B. A. Erwin J. P. Fleisher L. A.. (2017). 2017 AHA/ACC Focused Update of the 2014 AHA/ACC Guideline for the Management of Patients With Valvular Heart Disease. J. Am. Coll. Cardiol. 70 (2), 252–289. 10.1016/j.jacc.2017.03.011 28315732

[B34] Pasini E. Aquilani R. Testa C. Baiardi P. Angioletti S. Boschi F.. (2016). Pathogenic Gut Flora in Patients With Chronic Heart Failure. Jacc-Heart Failure 4 (3), 220–227. 10.1016/j.jchf.2015.10.009 26682791

[B35] Pihlstrom B. L. Michalowicz B. S. Johnson N. W. (2005). Periodontal diseases. Lancet 366 (9499), 1809–1820. 10.1016/s0140-6736(05)67728-8 16298220

[B36] Reményi B. Wilson N. Steer A. Ferreira B. Kado J. Kumar K.. (2012). World Heart Federation criteria for echocardiographic diagnosis of rheumatic heart disease—an evidence-based guideline. Nat. Rev. Cardiol. 9 (5), 297–309. 10.1038/nrcardio.2012.7 22371105 PMC5523449

[B37] Russell E. A. Walsh W. F. Reid C. M. Tran L. Brown A. Bennetts J. S.. (2017). Outcomes after mitral valve surgery for rheumatic heart disease. Heart Asia 9 (2), e010916. 10.1136/heartasia-2017-010916 29467839 PMC5818046

[B38] Smith D. A. Nehring S. M. (2020). “Bacteremia,” in StatPearls (Treasure Island, FL: StatPearls Publishing LLC).

[B39] Soderholm A. T. Barnett T. C. Sweet M. J. Walker M. J. (2018). Group A streptococcal pharyngitis: Immune responses involved in bacterial clearance and GAS-associated immunopathologies. J. Leukocyte Biol. 103 (2), 193–213. 10.1189/jlb.4MR0617-227RR 28951419

[B40] Tandon R. Sharma M. Chandrashekhar Y. Kotb M. Yacoub M. H. Narula J. (2013). Revisiting the pathogenesis of rheumatic fever and carditis. Nat. Rev. Cardiol. 10 (3), 171–177. 10.1038/nrcardio.2012.197 23319102

[B41] Tang W. H. W. Backhed F. Landmesser U. Hazen S. L. (2019). Intestinal Microbiota in Cardiovascular Health and Disease. J. Am. Coll. Cardiol. 73 (16), 2089–2105. 10.1016/j.jacc.2019.03.024 31023434 PMC6518422

[B42] Tompkins D. G. Boxerbaum B. Liebman J. (1972). Long-term prognosis of rheumatic fever patients receiving regular intramuscular benzathine penicillin. Circulation 45 (3), 543–551. 10.1161/01.Cir.45.3.543 5012243

[B43] Toscano M. De Grandi R. Stronati L. De Vecchi E. Drago L. (2017). Effect of Lactobacillus rhamnosus HN001 and Bifidobacterium longum BB536 on the healthy gut microbiota composition at phyla and species level: A preliminary study. World J. Gastroenterol. 23 (15), 2696–2704. 10.3748/wjg.v23.i15.2696 28487606 PMC5403748

[B44] Trompette A. Gollwitzer E. S. Yadava K. Sichelstiel A. K. Sprenger N. Ngom-Bru C.. (2014). Gut microbiota metabolism of dietary fiber influences allergic airway disease and hematopoiesis. Nat. Med. 20 (2), 159–166. 10.1038/nm.3444 24390308

[B45] Walker M. J. Barnett T. C. McArthur J. D. Cole J. N. Gillen C. M. Henningham A.. (2014). Disease Manifestations and Pathogenic Mechanisms of Group A Streptococcus. Clin. Microbiol. Rev. 27 (2), 264–301. 10.1128/cmr.00101-13 24696436 PMC3993104

[B46] Wang W. Chen L. Zhou R. Wang X. Song L. Huang S.. (2013). Increased Proportions of Bifidobacterium and the Lactobacillus Group and Loss of Butyrate-Producing Bacteria in Inflammatory Bowel Disease. J. Clin. Microbiol. 52 (2), 398–406. 10.1128/jcm.01500-13 24478468 PMC3911339

[B47] Wang L. Liu L. Liu X. Xiang M. Zhou L. Huang C.. (2020). The gut microbes, Enterococcus and Escherichia-Shigella, affect the responses of heart valve replacement patients to the anticoagulant warfarin. Pharmacol. Res. 159, 104979. 10.1016/j.phrs.2020.104979 32505835

[B48] Watkins D. A. Johnson C. O. Colquhoun S. M. Karthikeyan G. Beaton A. Bukhman G.. (2017). Global, Regional, and National Burden of Rheumatic Heart Diseas -2015. N. Engl. J. Med. 377 (8), 713–722. 10.1056/NEJMoa1603693 28834488

[B49] Watkins D. A. Beaton A. Z. Carapetis J. R. Karthikeyan G. Mayosi B. M. Wyber R.. (2018). Rheumatic Heart Disease Worldwide JACC Scientific Expert Panel. J. Am. Coll. Cardiol. 72 (12), 1397–1416. 10.1016/j.jacc.2018.06.063 30213333

[B50] Woldu B. Bloomfield G. S. (2016). Rheumatic Heart Disease in the Twenty-First Century. Curr. Cardiol. Rep. 18 (10), 11. 10.1007/s11886-016-0773-2 27566329 PMC9067597

[B51] Wong S. S. Y. Yuen K. Y. (2012). Streptococcus pyogenes and re-emergence of scarlet fever as a public health problem. Emerg. Microbes Infect. 1:10. 10.1038/emi.2012.9 PMC3630912 26038416

[B52] Yoshizawa J. M. Schafer C. A. Schafer J. J. Farrell J. J. Paster B. J. Wong D. T. (2013). Salivary biomarkers: toward future clinical and diagnostic utilities. Clin. Microbiol. Rev. 26 (4), 781–791. 10.1128/CMR.00021-13 24092855 PMC3811231

[B53] Zhang X. Zhang D. Jia H. Feng Q. Wang D. Liang D.. (2015). The oral and gut microbiomes are perturbed in rheumatoid arthritis and partly normalized after treatment. Nat. Med. 21 (8), 895–905. 10.1038/nm.3914 26214836

[B54] Zhang Y. H. Wang X. Li H. X. Ni C. Du Z. B. Yan F. H. (2018). Human oral microbiota and its modulation for oral health. Biomed. Pharmacother. 99, 883–893. 10.1016/j.biopha.2018.01.146 29710488

[B55] Zhang T. Kayani M. U. R. Hong L. Zhang C. Zhong J. Wang Z.. (2020). Dynamics of the Salivary Microbiome During Different Phases of Crohn’s Disease. Front. Cell Infect. Microbiol. 10, 544704. 10.3389/fcimb.2020.544704 33123492 PMC7574453

[B56] Ziebolz D. Jahn C. Pegel J. Semper-Pinnecke E. Mausberg R. F. Waldmann-Beushausen R.. (2018). Periodontal bacteria DNA findings in human cardiac tissue - Is there a link of periodontitis to heart valve disease? Int. J. Cardiol. 251, 74–79. 10.1016/j.ijcard.2017.09.001 29197463

