# Supplementary material for: Microbiota in Gut, Oral Cavity, and Mitral Valves Are Associated With Rheumatic Heart Disease
Source: Front Cell Infect Microbiol. 2021 Mar 9;11:643092. doi: 10.3389/fcimb.2021.643092 (PMC7985333; doi:10.3389/fcimb.2021.643092)
Supplement: Supplementary file 1 [file DataSheet_1.pdf]

Supplemental materials

Supplemental Table 1 Characteristics of the study subjects

Variables	Control subjects (n=20)	RHD patients (n=20)	p value
males, n (%)	9 (45)	7 (35)	0.5724
age, years	60.45±12.53	59.6±9.67	0.6436
aortic root dimensions, mm	30.75±3.338	31.3±2.736	0.5577
left atrial diameter, mm	36.5±5.031	53.75±10.15	<0.0001
inter-ventricular thickness, mm	9.5±1.235	9.8±1.642	0.3902
left ventricular posterior wall thickness, mm	9.25±1.251	9.7±1.72	0.1430
pulmonary trunk diameter, mm	23.25±1.446	24.2±2.238	0.1602
pulmonary artery systolic pressure, mm	35.67±7.678	46.84±13.4	0.0029
left ventricular ejection fraction, %	64.95±1.791	57.7±9.658	0.0031

Supplemental Figure 1

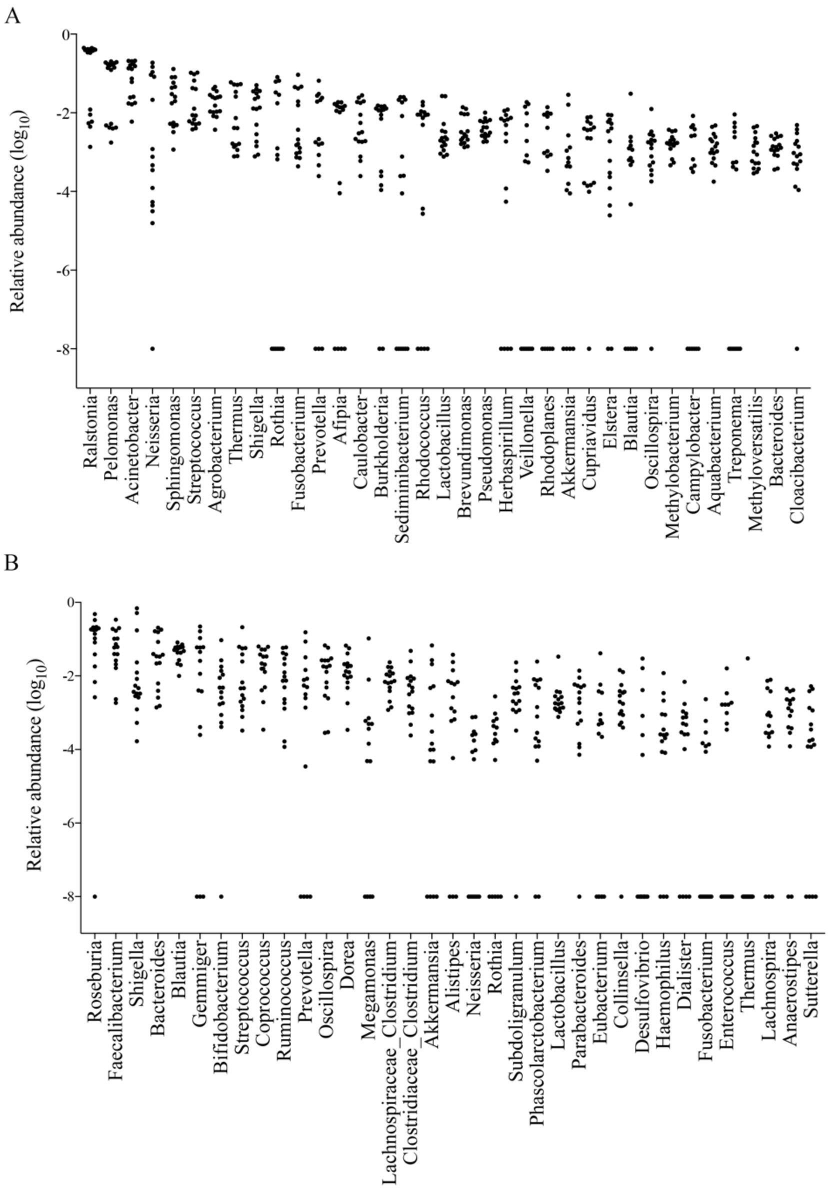

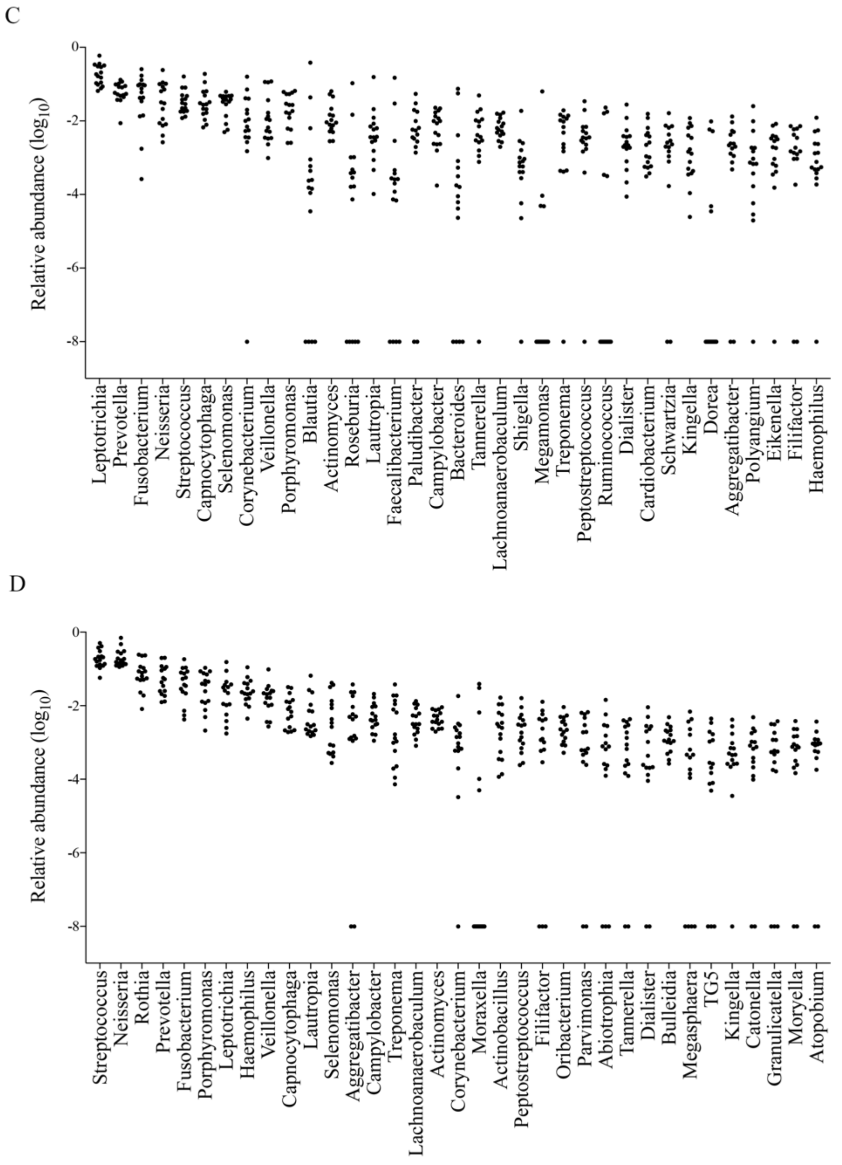

Supplemental Figure 1 Relative abundances of the 35 most abundant genera in microbiota of mitral valves (A), gut (B), subgingival plaques (C), and saliva (D) of patients with rheumatic heart disease.

Supplemental Figure 2

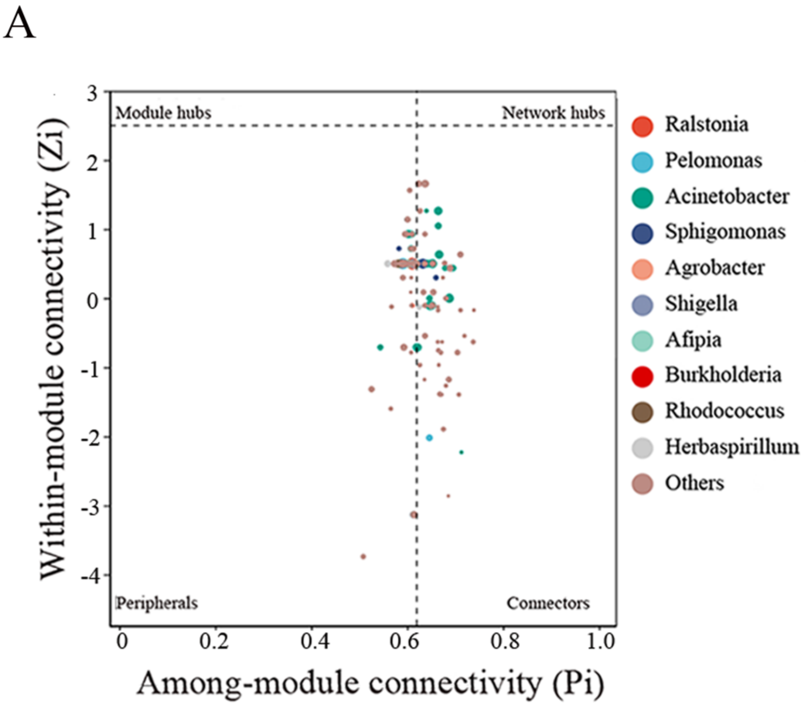

Supplemental Figure 2 Determination of module-based topological roles (peripherals, connectors, module hubs, or network hubs) of microbiota in mitral valves of patients with rheumatic heart disease using ZP-plot at genus level. The size of dots represents abundance of each genus.

Supplemental Figure 3

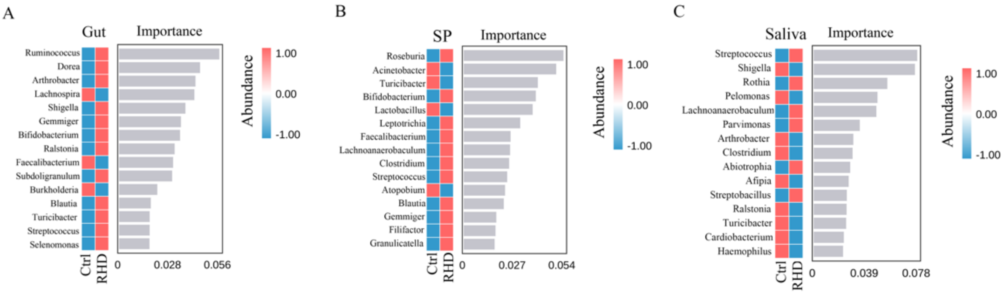

Supplemental Figure 3 Random forest algorithm-aided identification of genus classifiers and feature importance in gut and oral microbiota of patients with rheumatic heart disease (RHD) vs control subjects (Ctrl). Relative abundance and corresponding importance of the 15 most important genus classifiers in gut microbiota

(A), subgingival plaque microbiota (B), and salivary microbiota (C) are shown. SP indicates subgingival plaque.
